# New colonisers drive the increase of the emerging loggerhead turtle nesting in Western Mediterranean

**DOI:** 10.1038/s41598-024-51664-w

**Published:** 2024-01-17

**Authors:** Astrid Luna-Ortiz, Gisela Marín-Capuz, Elena Abella, José Luis Crespo-Picazo, Fernando Escribano, Guillem Félix, Silvia Giralt, Jesús Tomás, Cinta Pegueroles, Marta Pascual, Carlos Carreras

**Affiliations:** 1 Department of Genetics, Microbiology and Statistics and IrBio, University of Barcelona, Avinguda Diagonal 643, 08028 Barcelona, Spain; 2 BETA Technological Center, University of Vic - Central University of Catalonia, Carretera Roda 70, 08500 Vic, Spain; 3 Fundació Oceanogràfic de la Comunitat Valenciana, Ciutat de les Arts i les Ciències, 46013 Valencia, Spain; 4 Centro de Recuperación de Fauna Silvestre “El Valle”, Ctra. Subida a El Valle, 62, 30150 La Alberca de las Torres, Murcia Spain; 5 Consorci per a la Recuperació de la Fauna de les Illes Balears (COFIB), Servei de Protecció d′Espècies. Conselleria Agricultura, Pesca i Medi Natural. Govern de les Illes Balears, Carretera Palma- Sineu, Km 15,400, 07142 Santa Eugènia, Balearic Islands Spain; 6 Fundación para la Conservación y la Recuperación de Animales Marinos (CRAM), 08820 El Prat de Llobregat, Barcelona Spain; 7 Instituto Cavanilles de Biodiversidad y Biología Evolutiva, University of Valencia, Apdo, 22085, 46071 Valencia, Spain

**Keywords:** Population genetics, Evolutionary genetics

## Abstract

The loggerhead sea turtle (*Caretta caretta*) is sensitive to climate change and is responding by colonising the Western Mediterranean. To understand the rapid nesting increase in recent years in Spain, we sampled 45 hatchlings from 8 nests between 2016 and 2019. We sequenced a mtDNA D‐loop region, genotyped 2291 SNPs using 2bRAD and collected data on clutch size, hatching success, and incubation duration. We confirmed that the colonisation has a Mediterranean and Atlantic mixed origin and we detected that these nests were laid by different females, except for two nests within the same season. Our results suggest that the recent increase in nesting is due to an increase in the number of colonising individuals rather than females born in the same area returning to breed. We hypothesize that this increase in the number of colonisers results from successful conservation efforts, feminisation of the populations of origin and earlier sexual maturation. However, the percentage of offspring females produced in Spain suggests that future returning individuals will aid to the settlement of the new population. These results allow defining the current status of this colonisation although future efforts are needed to detect remigrants to confirm the establishment of a resident population.

## Introduction

Climate change is a major threat to global biodiversity^1^. The multiple components of this phenomenon are affecting directly all pillars of biodiversity and the species affected may respond in three ways: adapt, move, or become extinct. Some species are responding by shifting their geographic distribution or changing their phenology, altering their development and time of reproduction, modifying the composition of communities and their interactions^2,3^.Consequently, extinction can be avoided if populations move to favourable habitats, organisms successfully overcome stressful conditions via plastic changes, or populations undergo evolutionary adaptation^4,5^.

Among all marine species, sea turtles have a potential vulnerability to climate change, as multiple processes associated to this global phenomenon (e.g., increase of temperature, sea-level rise and increase of extreme meteorological events) can simultaneously affect these species during different stages of their lives and at large geographic scales^6,7^.The increase in temperature is thought to cause a great impact on sea turtles because they have life history traits strongly influenced by environmental temperature^8,9^. For instance, sand temperature during egg incubation plays a vital role in embryo development, hatching success, hatching sex ratio due to their temperature sex determination and post-hatchling fitness characteristic^10–12^. Consequently, increases in temperature may skew population sex ratios towards females^13^ collapsing the populations and compromising its long-term viability^14–16^. In addition, nesting beaches, especially reef islands, are likely to be impacted because of ocean acidification, affecting carbonate sediment production, sediment budget and sediment traits^17^. This potential alteration of the sediments is expected to affect sea turtles’ reproduction, as they require specific sediment characteristics to incubate their eggs and dig their nests^18^. Potential impacts range from changes in hatchling emergence success to loss of nesting habitat suitability^19^. For instance, the size of grains in the sand plays a crucial role in both gas exchange and the facility for hatchlings to emerge from their nests^18,20^. In addition, changes in grain size and sorting can affect sand temperature^21^, probably affecting sex determination. Considering all these potential impacts together, global warming is considered a major threat that jeopardises the viability of current nesting population^6,22^.

Under this scenario, the use of new nesting sites and the colonisation of new areas can be crucial for the survival of sea turtle species. Humans can favour this process with actions such as breeding individuals in captivity, reintroducing individuals in natural environments or restoring altered nesting areas. Some management actions have promoted the successful recovery of sea turtle nesting wild populations or the establishment of new populations through the release of individuals in new areas to overcome the putative constraints to colonise new areas imposed by philopatry^23,24^. However, the study and management of natural colonisations should be considered a priority for the species survival before using assisted colonisations^25^. In this sense, Carreras et al.^26^ analysed the sporadic nesting of the loggerhead turtle (*Caretta caretta*) in the Western Mediterranean and they discovered that these nesting events were due to colonisers from distant nesting areas in the western Atlantic and eastern Mediterranean and suggested that this natural colonisation was probably related to climate change. The authors predicted, using population modelling approaches, that this colonisation would raise rapidly under a global warming scenario. The increase of overall temperatures would favour the production of females in these new nesting events that would return to the new location when mature, in the process of becoming residents of the new population and reproduce in subsequent nesting seasons as remigrant. This colonisation would be later promoted by the philopatry of the species, as it implies that, once a sporadic nest is laid, the females born in this new nest would return to reproduce when they reach sexual maturity and might be detected in different nesting seasons (remigrant females)^27^. Consequently, detecting remigrant females nesting over subsequent nesting seasons would confirm the successful consolidation of a new population.

**Figure 1:**
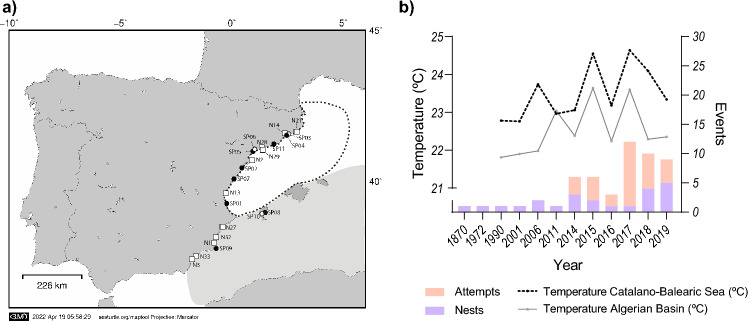
Summary of nesting activity in the Spanish coast. (**a**) Location of sporadic nests recorded in Spain from 1870 to 2019 (N = 22) as coded in Table 1 and Supplementary Table S1. Squares indicate nests laid between 1870 and 2015 in Spain as analysed in a previous study (N = 11)^26^. Circles indicate nesting events from 2016 to 2019 (N = 11) analysed in the present study, being the black circles, the nests analysed with 2bRAD sequencing (N = 8). Map created using MAPTOOL (SEATURTLE. ORG Maptool. 2002. SEATURTLE.ORG, Inc. http://www.seaturtle.org/maptool/ 18 Feb 2022)^97^. The two foraging areas in the region are highlighted with a black dashed line (Catalano-Balearic Sea) and a grey area (Algerian Basin). (**b**) Number of loggerhead turtle nests per year (N = 22) and attempts (N = 32) in the Spanish coast (Supplementary Table S1, Supplementary Table S2, adapted from Hochscheid et al.^28^. The lines represent the mean SST in June and July per year in the two foraging areas indicated in the map.

**Figure 2:**
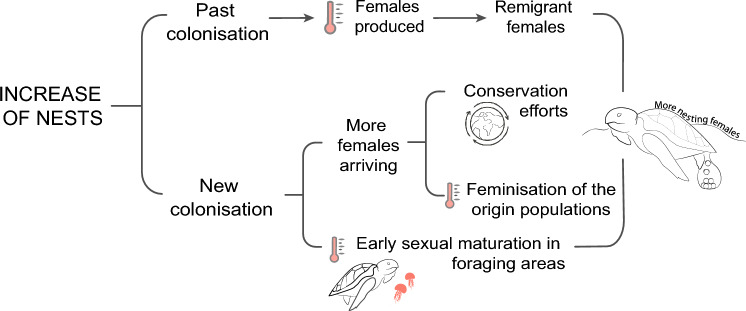
Non-exclusive hypotheses that may explain the increase in nesting activity in the Western Mediterranean basin. On one hand, females produced in past colonisation events could have returned upon maturation and established in the new areas as remigrant females. On the other hand, the increase of the nests could be related to an increase in the arrival of new colonisers. This increase could be produced by the arrival of new females, either due to the increase of the populations due to conservation efforts or due to the feminisation of the origin populations, of because of an early sexual maturation in foraging areas, increasing the chances of laying eggs outside of their origin nesting populations.

The predictions about a future increase of the nesting activity^26^ seemed to be accomplished as an unprecedented number of nests started to be reported in the Western Mediterranean in the last decade^28^, including the Spanish coast (Fig. 1a, b). This increase has implied that the nesting range of the species in the Mediterranean has been moving westwards associated to anthropogenic variables and sea surface temperature^29,30^. Two non-exclusive hypotheses can explain the increment of nesting activity in the western basin (Fig. 2). The first possibility is that the incipient population started to grow as the result of remigrant females that were born in the new location in the past that, after maturation, are currently returning to reproduce in the new area due to philopatry, as already detected in Conigli beach, in Lampedusa, Italy^26^. The second possibility is that the number of nests may be increasing because more colonising females are arriving at the new sites from their populations of origin. This increase in the number of arriving colonisers could be due to an increase in the number of females on the populations of origin, which might be a result of conservation efforts^31^, due to feminisation of the populations^13,32^, or due to both processes together. Another reason of the increase of the number of colonising nesting females could be related to an earlier maturation of the females in foraging areas related to an increase of sea temperature^29,33,34^.

Understanding the emergence of new nesting areas has a strong evolutionary and conservation importance as it provides the opportunity to study colonisation-in-action events of long living philopatric animals^26^. Tagging-recapture studies in marine turtles, using either physical^35^ or genetic tagging^36^, have shown that the degree of philopatry is variable among and within species, and that deviations of tens of kilometres between nesting activities may happen. However, the presence of these non-strictly philopatric individuals could only explain short distance migrations along continuous nesting habitats, due to the short dispersion range. Therefore, sea turtles should have a mechanism for long-distance colonisation, as indicated by the widespread distribution of the species around the world^37^, resulting from millions of years of evolution in environmental changing conditions. Likewise, the study of long-distance colonisation processes, such the one that is happening in the Western Mediterranean^26^, is key to understand the origin and significance of both new and historical nesting events in sea turtles and to establish conservation plans in the context of the current global warming.

Research on marine turtles is challenging but studying an ongoing colonisation of marine turtles poses additional limitations. Scientific methods commonly used in regular nesting areas cannot be directly applied in this case due to the scattered distribution of nests along large coastlines^28^. In this context, genetic tools have been employed to gather reliable biological information from a limited number of samples when nesting is scarce, particularly in cases when detecting nesting females proves challenging^36^. However, genetic markers such as mitochondrial genes are limited when assigning nests to specific females or inferring the adult breeding population^26^. Nuclear markers are useful for this purpose, but the number of markers is a key factor in the analysis of genetic differentiation^38^. Thus, genomic methods are preferred when studying newly colonised marine turtle nesting sites. Due to the thousands of loci recovered with these techniques, thorough analysis is possible with only a few samples, providing essential information for conservation purposes^39^.

In the present study we aim to understand the phenomenon of the long-distance dispersal and colonisation to new suitable shores by loggerhead turtles, testing the hypotheses that have been suggested (Fig. 2) to explain the recent increase in the nesting events in the Western Mediterranean. To do so, we explored the genetic composition of sporadic nests laid on the Mediterranean coast of Spain from 2016 to 2019 and we combined this genetic data with reproductive and environmental information collected in the nesting locations. Besides the inherent scientific interest, this work will be of importance when designing new conservationism protocols to aid the establishment of the loggerhead turtle on Spanish coasts.

## Results

In Spain’s Mediterranean coasts, the number of loggerhead turtle nesting events have been increasing since 2001^40^ and since 2014 the species is nesting annually showing an increasing trend^28^ (Fig. 1b). We gathered information on the 11 nesting events recorded over 2016–2019 years (Table 1, Supplementary Table S1), obtaining detailed data on all of them except from nests SP02, SP06, and SP11 as only hatchlings on the beach were found and clutches were not found. The mean clutch size per nest was 97 eggs (SD ± 35.11) and all nests but one (SP10) yielded viable hatchlings with hatching success ranging between 0% and 93.1%, with a mean of 55.56% (SD ± 29.12), showing also a high variability across nests (Supplementary Table S1). All clutches were laid in the summer season, between the months of July and August and hatched between the months of August and October (Supplementary Table S1). Rates of female offspring obtained from incubation durations ranged from 0 to 100%, with some variability within nest depending on the models used (Supplementary Table S1). We found a female-biased sex ratio in all but two of the nests analysed in this study (SP01 and SP07) that showed a male biased sex ratio. The habitat suitability of the nesting locations, according to estimated published map models for the Mediterranean region based on nine independent terrestrial temperature and precipitation variables^41^, categorized the locations as “Marginal”, “Good” and “Excellent”^41^, and with a generally predicted to increase in hatching success in the future^42^ (Supplementary Table S1).

**Table 1:** Sporadic nests of loggerhead turtle in the Spanish coast from 2016 to 2019 and genomic data associated.

ID	Year	*Ld*	*Ed*	Beach (Locality)	Samples analysed	mtDNA		Polymorphic nuclear loci (%)	Mean Ho ± SD	Mean relatedness
						Haplotype	Haplotype origin			
SP01	2016	03/07/2016	05/09/2016	Les Palmeres (Sueca)	2	CC-A2.1	Shared	43.39	0.255 ± 0.01	0.082
SP02	2017	N/A	11/10/2017	Migjorn (Peñiscola)	2	CC-A1.1	Atlantic	38.19	0.253 ± 0.006	0.180
SP03	2018	15/06/2018	08/08/2018	Sant Simó (Mataró)	–	–	–	–	–	–
SP04	2018	01/08/2018	28/09/2018	La Descàrrega (Premià de Mar)	8	CC-A3.1	Shared	48.8	0.253 ± 0.008	0.235
SP05	2018	N/A	16/09/2018	Vilafortuny (Cambrils)	4	CC-A2.1	Shared	43.3	0.275 ± 0.035	0.301
SP06	2018	N/A	24/09/2018	Ardiaca (Cambrils)	–	–	–	–	–	–
SP07	2019	13/07/2019	14/09/2019	Del Serradal (Castellón de la Plana)	7	CC-A31.1	Mediterranean	49.24	0.279 ± 0.046	0.229
SP08	2019	25/07/2019	10/09/2019	D’en Bossa (Sant Jordi de ses Salines)	7	CC-A2.1	Shared	52.68	0.317 ± 0.051	0.221
SP09	2019	28/07/2019	18/09/2019	Calblanque (Cartagena)	7	CC-A2.1	Shared	50.72	0.283 ± 0.045	0.216
SP10	2019	29/07/2019	N/A	D’es Cavallet (Sant Francesc de s’Estany)	–	–	–	–	–	–
SP11	2019	N/A	06/10/2019	Castelldefels (Castelldefels)	8	CC-A31.1	Mediterranean	47.4	0.243 ± 0.006	0.225

*Ld*, Date of egg laying; *Ed,* date of first emergence; mtDNA, nest haplotypes and attributed nesting area to each haplotype; Polymorphic loci, percentage of polymorphic nuclear markers per nest; *Ho*, observed heterozygosity; Mean relatedness, relatedness per nest inferred using Manichaikul relatedness index^44^. N/A indicates data is not available. A value of (–) indicates that no genetic data could be obtained from these nests. Additional information of these nests can be found in Supplementary Table S1.

### SST in surrounding foraging areas during nesting period

**Figure 3:**
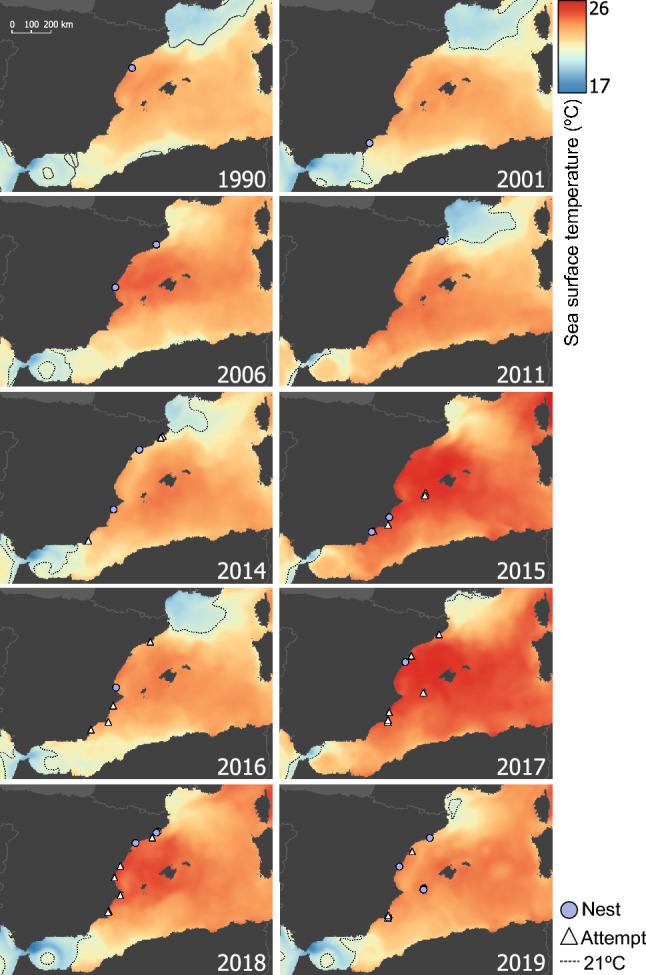
Spatial distribution of events detected by year (1990–2019) and the corresponding SST mean temperatures between June and July for each year. Only the SST gradient of the years with presence of nesting events (light blue circles) or attempts (white triangles) is illustrated. The isotherm of 21 °C SST, which is the threshold suggested as critical for the presence of nesting events in the Western Mediterranean^34^ is represented with a dashed black line. Maps created using QGIS vs 3.22.9 software (https://www.qgis.org/en/site/).

All events that occurred between 1990 and 2019 in the Spanish coast (including attempts and nests, Supplementary Table 1–2) were in areas with a sea surface temperature (SST) above 21 °C (Fig. 3), which is considered the temperature when the nesting season starts^34^. We detected a significant correlation between the annual number of total events in the Spanish coast (Supplementary Table 1–2) and the mean SST in the same nesting period in the Catalano-Balearic region (Spearman’ rank correlation, ρ = 0.825; p-value = 0.003), but not the Algerian Basin mean SST (Spearman correlation, ρ = 0.554; p-value = 0.096).

### Genetic analyses

We obtained genetic data from 45 samples from 8 of the 11 nesting events that occurred from 2016 to 2019 nesting seasons. DNA extractions from samples of nest SP03 failed due to bad preservation conditions, no development was observed for any of the eggs of nest SP10 (Supplementary Table S1) and no samples were collected from nest SP06 by the authorities that attended these nesting events, although free hatchlings were observed on the beach and no clutch was found.

We obtained a total of 4 different D-Loop mtDNA haplotypes in the 8 nests (Table 1), all of them previously found in other areas^43^. The samples from 5 nests had haplotypes that can be found both in the Atlantic and in the Mediterranean nesting beaches (CC-A2.1: SP01, SP05, SP08, SP09; CC-A3.1: SP04). One nest had a haplotype that is exclusive to the Atlantic nesting populations (CC-A1.1: SP02), while the remaining two nests shared a rare haplotype that has been only found in Mediterranean nesting beaches (CC-A31.1: SP07 and SP11).

With 2bRAD sequencing we obtained a total of 246,481,025 raw reads (Supplementary Table S3), with an average and standard deviation of 5,477,356.11 ± 1,583,759.95 reads per individual. The percentage of mapping to the reference loggerhead genome was on average (SD) 93.8% (6.3) (Table S3). We detected a total of 154,613 non-filtered SNPs across all samples. After applying all filters and selecting only loci shared by at least 95% of the individuals, we retained a total of 2,291 loci, with an average depth of 25.07 reads per locus.

**Figure 4:**
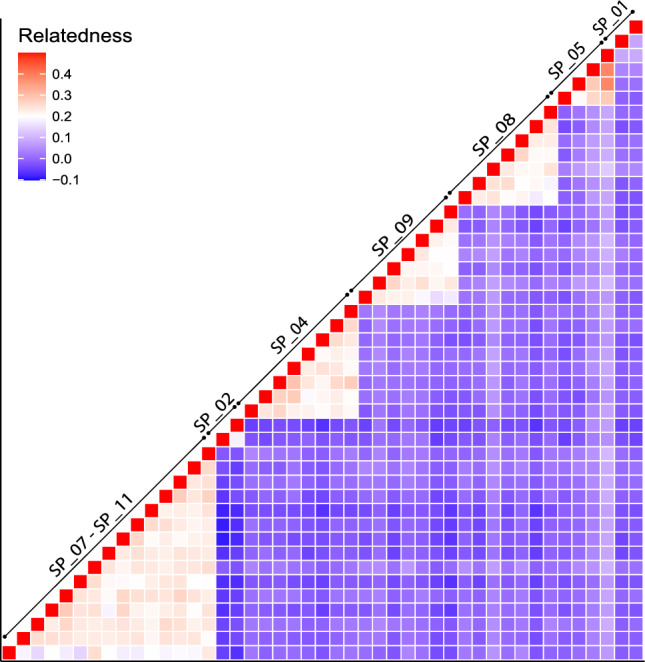
Heatmap based on the relatedness index among pairs of individual samples based on the 2,291 filtered SNPs obtained with 2bRAD sequencing. Each cell represents the value of the Manichaikul relatedness index^44^ between sample pairs. The samples that belong to each nest are indicated in the diagonal arrows. Individuals from nests SP_07 and SP_11 clustered together. Nests are coded as in Table 1.

**Figure 5:**
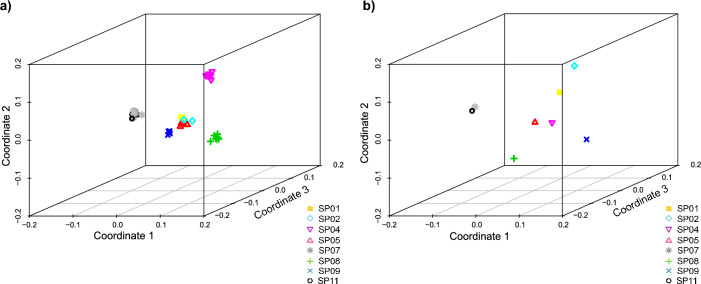
Multidimensional scaling (MDS) analysis plots based on IBS distance among the 45 individuals included in this study (2,291 SNPs), representing the genetic differentiation of the 8 nests analysed with 2bRAD sequencing on the Spanish coast. (**a**) 3D scatter plot considering all the individuals (N = 45). (**b**) 3D scatter plot, considering one individual per nest (N = 8). Nests are coded as in Table 1.

The percentage of polymorphic loci in each nest was variable (mean 46.72% of the loci; SD ± 4.75, Table 1). The observed heterozygosity value across all samples was similar (mean Ho = 0.271 ± SD 0.04, Supplementary Table S3), and the same was true for the observed mean heterozygosity per nest (mean Ho = 0.270 ± SD 0.02, Table 1). The relatedness^44^ values between individuals of the same nest were variable across nests (ranging from 0.082 to 0.3; mean 0.211; SD ± 0.062, Table 1), but generally much higher than between individuals from different nests (Fig. 4). The comparison of genotypes allowed us to identify re-nester females, defined as females laying multiple clutches, that could also be considered remigrant females if these clutches were laid in different years. Only in one case, the individuals from two different nests clustered together with relatedness values (nests SP07 and nest SP11), thus suggesting that the same female laid both nests (Fig. 4). Moreover, the pair of individuals from the nest SP01 presents lower relatedness values than other nests, suggesting possible multiple paternity within this nest (Fig. 4) as found in previous studies in the region^26^. Additionally, the MDS plot based on IBS distances generally clustered all the individuals from the same nest when considering the first three-axis while separated individuals from different nests (Fig. 5a). All individuals from the nests SP07 and SP11 clustered together with all three axes. However, three of the nests (SP01; SP02; SP05) also clustered in the centre of the three axes despite their low relatedness values (Fig. 4) and the different potential origin of the D-loop haplotypes, exclusive of the Atlantic for SP02 and shared among basins for SP01 and SP05 (Table 1). When only one randomly picked individual per nest was used to build the MDS plot, in order to avoid artificial clustering related to uneven sampling size, no clustering was observed except samples from the nests SP07 and nest SP11 (Fig. 5a). Interestingly, SP02 with a characteristic Atlantic D-loop haplotype was clearly separated by the first coordinate (Fig. 5b).

## Discussion

Sea turtles are currently facing several climate warming impacts, ranging from rising of sea level to the thermal increase of ocean water and incubation conditions^45^ which are predicted to be especially severe in the Mediterranean Sea^46,47^. As a probable adaptive response to the increased temperature, the loggerhead sea turtle is colonising the Western Mediterranean^26,29,48,49^. The nesting activity on the beaches in the Western Mediterranean has experienced an explosive increase in the last decade^28^ with nesting becoming regular in some regions of Italy^48,49^ suggesting that this colonisation process has started to consolidate^28^. By using genetic tools and in situ data collection, our results show that this emerging colonisation in Spain increased in numbers by non-related females laying eggs in different years, rather than because of the presence of remigrant females. However, considering the abundance of females produced because of the incubation conditions in the nests, there is potential to find remigrating females in the near future indicating the settlement of the new population.

In the present work, viable hatchlings were produced in most of the nests although with a high variability among nests (from 0 to 93.1% of hatching success), as reported in previous years in the Western Mediterranean region^26,28^. The reasons of this variability may be caused by nonexclusive factors, including local environmental conditions, different management strategies, or biological factors (body conditions of the female or genetic factors of both parents). Future research in emerging nesting sites is needed to unveil the reasons for these differences. A recent study analysing past temperature records has shown that the annual window for viable nesting in the Western Mediterranean (defined as the temporal window in which sand temperatures are suitable to host a viable nest during all its incubation) was small or absent until recently due to the cold-water temperature but has increased over the past ten years^34^. If temperatures continue rising in the Mediterranean^1^, the habitat suitability for loggerhead nesting will increase in the Western Mediterranean^45^, but also the nests laid in these areas will produce a higher proportion of females^26,33^. Our results regarding the inferred sex ratio are in line with what has been found in other areas of the central and western Mediterranean^28,50^, with a combination of nests with a high proportion of females but also some nests with a male biased sex ratio. Hence, given the natural philopatry of the species^33^, the high percentage of female offspring could result in a potential increase of individuals returning as breeding adults, consolidating the establishment of the new population under suitable environmental conditions^26^.

Our results also confirmed that the colonising individuals come from distant regular nesting areas in the Western Atlantic and eastern Mediterranean Regional Management Units (RMU)^37^, as found in older nests^26^. The sequencing of the mtDNA D‐Loop region revealed three nests that presented a haplotype exclusive from an RMU. Two of these nests (SP07-SP11) presented a haplotype (CC-A31.1) previously found in three non-related nests in Calabria and Kyparissia, regular nesting populations from the eastern Mediterranean^51^, and in a nest from the Ionian coast of Sicily in 2010^52^. Additionally, the nest (SP02) presented a haplotype (CC-A1.1) that is exclusive from the Atlantic and widespread in almost all Western Atlantic populations^43^. The remaining five nests with genetic data presented haplotypes with a shared origin between both the Western Atlantic and Mediterranean regular nesting areas. These results align with previous studies^26^, confirming a mixed presence of colonisers from different populations in the Western Mediterranean, and is consistent with the presence of juveniles and subadults from both regular nesting regions in nearby foraging areas^26,53^. Considering that the Atlantic and Mediterranean regular nesting areas are genetically very different and until now remained isolated^54^, this bilateral colonisation may lead to a novel genetic admixture. This potential admixture can increase the fitness of the offspring because of the hybrid vigor, but it can also produce the opposite effect due to outbreeding depression^55,56^. Consequently, knowing the origin of the nests and assessing the degree of admixture between Atlantic and Mediterranean individuals is crucial to understand the genetic viability of the emerging population, especially considering the high variability found in terms of hatching success. Genomic methods allow for studying migration patterns^57^ and population structure^58^, thus tracing the origin of the breeding individuals. Unfortunately, all these analyses require the establishment of a baseline by characterising the regular nesting areas with the same markers used in the emerging nesting sites, something not yet available with the genomic markers used in the present study but published for the mtDNA D-loop region analysed^43^. For this, and considering the explosive increase of the nests, a baseline of genomic data built with individuals from different regions is in progress to improve the origin assignment and admixture in future studies.

The recent increase in nesting activity in the Western Mediterranean^28^ and the higher production of females, at least in some nests (present study), indicate that we may be at the beginning of the establishment of the new population. Previous research suggested that the new population would start to grow exponentially once females born in the new area start to return due to philopatry and reproduce in different nesting season as remigrants^26^. Consequently, the detection of returning nesting females in different nesting seasons would be indicative that the colonisation in our study area has reached this point. Our MDS results based on 2,291 SNPs, clustered individuals from different nests that could suggest that were laid by the same re-nesting female. Individuals from the nests SP01, SP02 and SP05 clustered together in the MDS and were laid in 2016, 2017 and 2018 respectively. However, we can discard that some of these nests were laid by the same female as different mtDNA haplotypes were found in different nests (CC-A1.1 in SP02 and CC-A2.1 in SP01 and SP05) and were clearly separated by the relatedness analysis (e.g., individuals from different nests exhibited very low relatedness values). In addition, we discarded the presence of remigrant females since none of the individuals analysed clustered with individuals from different years, therefore suggesting that nests were laid by different females. Although, analysing a single individual per nest could be enough to perform a MDS to detect clusters of different nests, the genomic analysis of multiple individuals per nest allowed us to identify an artifact caused by uneven sample size. The MDS with a single individual per nest (Fig. 5b), splits the individuals from nests SP01, SP02 and SP05, especially the individual from nest SP02 with a D-loop haplotype widespread in the Western Atlantic rookeries. Additionally, analysing multiple individuals per nest helps delineating clusters of siblings to the same nest in relation to individuals from other nests with a relatedness analysis. Consequently, the number of related samples that are included when using all data together must be evaluated depending on the analysis performed. Thus, the similarity between related samples and uneven sampling sizes may distort the relationships with the rest of the samples and may affect the interpretation of the results of the MDS as the presence of many related individuals seems to mask the relationships between unrelated ones. So, the number of samples from the same nest or the same area that are included in the analysis when analysing all data together may affect the MDS plot and should be considered in genetic analyses to correctly identify the relationships.

The hatchlings from nests SP07 and SP11 clustered together in the MDS, have the same rare mtDNA haplotype and exhibited high relatedness values to the point that relatedness among individuals from these two nests were indistinguishable from siblings within the same nest. All this evidence supports the fact that only these two nests were laid by the same re-nesting female, the same year and 213 km apart. The exact laying date of nest SP11 is not available, as hatchlings were detected during the emergence phase. However, considering the range of incubation duration in our data, the female laid the two nests in an interval between 14 and 30 days, consistent with the internesting interval in loggerhead turtles which is between 12 and 16 days^59–62^. The detection of a re-nesting female laying more than one nest within the same year is not unprecedented, as previous studies on the loggerhead sporadic events in the Western Mediterranean, found one female laying two nests in 2015 in a 14-day interval and 120 km apart^26^. In regular nesting areas, loggerhead turtle females lay on average 3–5 nests per season with the internesting intervals mentioned above^63^. On green turtles the number of nesting events per season can be even higher and at shorter internesting intervals^25^. Multiple nests per female within the same year are the consequence of the gradual maturation of eggs, and even can be the result of the same mating event^64,65^. Thus, the nests laid by a female within the same year are considered part of the same reproductive season, since usually the female remains in the area or performs short internesting migrations during the whole nesting season^66^. Alternatively, if we had found nests laid by the same female in different years, this would imply that it is a remigrant due to philopatry, which in marine turtles involve long recurrent migrations from breeding to foraging areas^25,67^.

While nesting activity is becoming more frequent on the Spanish Mediterranean seaside, our results and those obtained by Carreras et al. 2018 suggest that this increase is not the result of remigrant individuals but an increase of the number of colonisers coming from distant areas. On one hand, both studies concluded that no remigrant was present in all nests analysed within their respective periods. On the other hand, despite both studies used different markers, they both analysed the same region of the D-loop, which is not very informative as common haplotypes are very frequent. Consequently, we cannot rule out that common haplotypes between both studies correspond to the same remigrating female, but different haplotypes are an indication of different nesting females. Considering that the typical remigrant interval of the species is two years, only one nest laid in 2014 had the same haplotype of the nest laid in 2016. On the contrary, no nest laid in 2015 had the same haplotype than the nest laid in 2017. These two-year comparisons suggest that nests among different reproductive seasons were laid by different females with only one potential remigrant across studies (Supplementary table S1). Future studies analysing all the sporadic events with the same methodology are desirable to confirm our hypothesis, particularly if the nest laid in Tarragona in 2014 (N29, CC-A2.1) was not laid by the same female than the nest laid in Sueca in 2016 (SP01, CC-A2.1), but also testing all potential pairs of nests in case there are individuals remigrating at longer intervals in this emerging population. An additional consideration is that the present study includes the nests detected over the four nesting seasons, but some nests may remain undetected and thus unsampled. Consequently, there is still the possibility that remigrants could be present in the unsampled nests. Considering all the evidence together and the potential drawbacks of the study, we propose that the recently raised number of nests may be caused by two non-exclusive hypotheses (Fig. 2). On one hand, the number of females arriving to the Western Mediterranean as potential colonisers may be increasing. This could be favoured by the global increase in the size of the sea turtle regular nesting populations^31^ derived from the success of conservation efforts worldwide^68–70^. Furthermore, we are witnessing a feminisation of sea turtle populations^31^ triggered by climate change^71^ which would drive an increase in the number of females recruiting to the adult population and a rise in the number of nests^72^ even with the same total number of adult individuals. Thus, the feminisation of the populations of origin would likewise increase the number of prospective colonising females found nesting in the emerging nesting sites. On the other hand, the increased sea surface temperatures in the Western Mediterranean might be favouring an early maturation of the juvenile or adult females feeding in the neighbouring foraging grounds^33^, as SST in feeding areas affects nesting phenology, leading to an earlier onset of nesting^73^. This early maturation would increase the chances of laying a nest in the nearby area, as this advanced maturation is produced before they are able to return to the nesting beaches of origin to reproduce^29^. Previous studies indicated that a minimum sea surface temperature of around 22 °C is needed for gonad maturation^74,75^ and 21 °C are needed to initiate the nesting season^34^. Our results show that the spatial and temporal location of the nesting events in the Spanish Mediterranean coast has always been above this threshold during the nesting years. Hence, the combination of the factors described above may be increasing the number of mature females in the Western Mediterranean thus explaining the current explosion of the number of clutches.

Our results confirm that we are witnessing a shift of species distribution at evolutionary level induced by climate change. Conservation measures are essential to help the growing population. First, monitoring the different approaches of this event is needed to evaluate the effects of rising temperatures on sex ratio, fitness, viability, and hatchling survival. Second, as emerging nests are occurring on anthropized beaches, analysing the human impact on the potential nesting beaches is essential to ensure the minimal anthropic disturbance. For instance, the effects of light pollution^76^ or the coastal erosion from massive urbanization^77^. Furthermore, education and awareness are fundamental parts of this framework. Citizens play a key role in the detection of the nests, which is substantial to obtain samples and biological data and ultimately, enabling management. Indeed, this study has added significance by becoming a pilot project for future colonisation events in migratory species influenced by human pressures. Translation of scientifically based monitoring to proactive conservation measures could facilitate the expansion and viability of the species in a warming world. The detection and study of these new events through extensive genomic monitoring and study on potential suitable habitats, coupled with its protection and conservation may be crucial to facilitate the possible expansion and long-term survival of the species.

## Methodology

### Sampling and data collection

Data and samples were obtained from loggerhead sea turtle nests laid on the Spanish coast between 2016 and 2019 (Table 1). When a nesting event occurred, we collected the basic data, previously published^28^. In addition, we estimated the minimum and maximum percentage of female offspring using the incubation duration and applying four different models^78–81^. We also gathered information on the habitat suitability for nesting^41^ and the present and future hatching success^42^ from published map models following the same procedures of previous studies^26^. Although the study species is listed in CITES, transportation of samples within the same country does not require CITES permits. As part of the national and regional management plan of the nesting events, some of the hatchlings are routinely kept for one year as part of a headstarting program to increase their first-year survival in the Foundation for the Conservation and Recovery of Marine Animals in Barcelona (CRAM), the Oceanografic of Valencia or the Palmaquarium Foundation of the Balearic Islands. Taking advantage of this management action, we obtained blood samples from headstarted individuals from some of the nests (SP04; SP07; SP08; SP09; SP11) taken at approximately one year of age as part of their routinely veterinary check. Approximately, 100 µl of blood was extracted from the cervical sinus following standard procedures^82^. Additionally, muscle or skin samples were collected from dead hatchlings or embryos found during the nest excavation after natural emergence of hatchlings. Both blood and tissue samples were fixed and stored in 96% ethanol at − 20 °C.

### Laboratory procedures

Genomic DNA was extracted using the Puregen Kit (Qiagen) following the manufacturer's protocol and suspended in 25 µl of Elution Buffer, the DNA concentration and quality was measured with Nanodrop. We considered one sample per nest for sequencing an 800‐bp fragment of the mtDNA D‐Loop region^83^. Each reaction was prepared in a final mix volume of 15 µl containing 5.08 µl of Nuclease Free-water (Thermo Scientific), 3 µl of PCR Buffer 5X (GoTaq Promega), 1.8 µl of dNTPs (1 mm), 0.6 µl of MgCl_2_ (25 mm), 1 µl of Forward primer (10 µm), 1 µl of Reverse primer (10 µm), 0.12 µl of Gotaq G2 Flexi DNA Polymerase (Promega 5U/µl), and 2 µl of DNA (~ 10 ng/µl), the amplified was verified in a 1% agarose gel. Next, 3 µl of the amplified product were purified with 2 µl of ExoSAP (0.4 U of EXO and 0.4 U of TSAP). Later, 1 µl (5 µm) of Forward primer was added to the purified product and dried at 80 °C for 30 min. We used only the Forward primer, as it was sufficient to recover the entire ~ 800 bp sequence used for haplotype delimitation for comparison in public databases. Finally, the amplification was sequenced on an ABI 3730 automated DNA analyser (Applied Biosystems) at the Seveis Cientificotècnics from the University of Barcelona.

To perform a 2bRAD genotyping, we analysed a variable number of individuals per nest (Table 1), depending on the availability of samples. We constructed individual libraries digesting 180 ng of DNA (~ 40 ng/µl) with Alfl enzyme using the protocol from Barbanti et al. (2020)^38^. The quantity and concentration used maximizes the total number of sequences recovered, as shown in previous studies^39^. For this study, we used selective-adaptors (5’-WN-3’) that reduce the number of analysed markers without compromising genetic differentiation^84^. After digestion, ligation and DNA amplification, the quality of the amplified fragment (~ 165 bp) was verified in a 1.8% agarose gel. The successful amplified product was purified using magnetic beads (SPRIselect) to remove primers and fragments longer and shorter than 165 bp. The DNA concentration of the purified libraries were measured with the Quant-iT™ Picogreen dsDNA Assay Kit (Thermo Fisher Scientific) and pooled calculating ~ 180 ng of DNA per sample. The pool was sequenced with a HiSeq 2500 Ilumina at the Centre for Genomic Regulation (CRG).

### Data filtering and genotyping

We processed the 2bRAD sequences using customized scripts^39^, trimming the raw sequences to remove ligation adaptors and cutting all the fragments to the same length (34 bp). We mapped the trimmed sequences to the published reference genome of the loggerhead turtle (GenBank accession GCA_023653815.1)^85^ using Hisat2-2.2.1^86^, to identify polymorphic nucleotides (SNPs) with BCFtools^87^. Individual genotypes were outputted as SNPs in a VCF file. We filtered our data using VCFtools^88^, by removing individual genotypes based on less than five reads. Loci with a mean depth above 50 (which corresponds to the upper whisker defined as 1.5 times the interquartile range from the data) and loci present in less than < 95% of the individuals were removed.

The D-Loop sequences were aligned, cut and blasted with published haplotype sequences found in the database maintained by the Archie Carr Center for Sea Turtle Research (https://accstr.ufl.edu/) using BIOEDIT^89^. We identified the regular nesting region in which each haplotype was found following the haplotype frequencies found in previous studies^43,90^ to determine the potential origin of the nesting females that laid each nest.

### Genetic diversity and relatedness

The percentage of polymorphic loci and observed heterozygosity per nest were used as a measure of relative genetic diversity. The percentage of polymorphic loci per nest was calculated using GENALEX 6.503^91^ and the observed heterozygosity was obtained using the function ‘–het’ of VCFTOOLS. Relatedness among individuals was calculated based on the number of alleles shared between pairs of samples by applying the ‘–relatedness’ statistic function of VCFTOOLS based on the Manichaikul et al.^44^ method. We used these relatedness values to create a heatmap and a dendrogram using the function ggplot of ‘ggplot2’^92^ in R program vs 4.1.1^93^. Finally, we used PLINK vr. 1. 07^94^ to perform a Multidimensional Scaling Analysis plot (MDS) considering the Identity By State (IBS) individual pairwise distance and the first 3 dimensions were plotted using the function scatterplot3d of ‘ggplot2′^92^ in R program vs 4.1.1^93^. Likewise, we also randomly selected one individual from each relatedness cluster, to plot an additional three-dimensional MDS.

### SST during nesting period

Western Mediterranean SST values were obtained from E.U. Copernicus Marine Service Information (doi.org/10.48670/moi-00173) for the months of June and July in the years when nesting or attempted nesting occurred between 1990 and 2019. These months were selected following the same rationale than previous studies^34^ since the nesting in the western Mediterranean is concentrated around this period (Table S1), and it has been suggested that a minimum of 21 °C of SST is needed to initiate nesting^29,34^. For each year, we obtained the mean daily SST of the two months considering two separated areas, on one hand the Balearic Sea (42.6 N, 39.1S, 4.2 E, − 0.5 W) and on the other the Algerian Sea (38.9 N, 34.8S, 6.6 E, − 2.4 W) as these areas are used by individuals foraging in the Western Mediterranean^53,95,96^. The daily mean SST for each area was averaged for every year using map algebra with the QGIS software vs 3.22.9. We used a Spearman’s rank correlation test (as a non-parametric analysis) to evaluate if the SST mean temperatures were correlated to the annual number of events, using the cor function as implemented in R program vs 4.1.1^93^. Finally, the fine scale SST spatial distribution of years with nesting events were graphically plotted through QGIS vs 3.22.9 software.

### Supplementary Information

Supplementary Information.

## Data Availability

D-loop haplotypes accession numbers are given in the results (CC-A1.1 EU179436; CC-A2.1 EU179445; CC-A3.1 EU179455; CC-A31.1 AM949678) and also can be found in the database maintained by Archie Carr Center for Sea Turtle Research (https://accstr.ufl.edu/). 2bRAD raw data can be found at the European Nucleotide Archive (ENA) project PRJEB64665. Data on the nesting events can be found in the Supplementary material.
