# Supplementary material for: New colonisers drive the increase of the emerging loggerhead turtle nesting in Western Mediterranean
Source: Sci Rep. 2024 Jan 17;14:1506. doi: 10.1038/s41598-024-51664-w (PMC10794258; doi:10.1038/s41598-024-51664-w)
Supplement: Supplementary file 1 — Supplementary Information. [file 41598_2024_51664_MOESM1_ESM.docx]

**Supplementary Tables**

| **Nest**  **code** | **Year** | **Beach (Location)** | **Description** | **Latitude [°N]** | **Longitude [°E]** | **Laying date** | **Emergence date** | ***Eg*** | ***Hs [%]*** | ***Id*** | ***Of [%]*** | **Habitat** | **Predicted Hs** | | | | **Haplotype** | **Reference** |
| --- | --- | --- | --- | --- | --- | --- | --- | --- | --- | --- | --- | --- | --- | --- | --- | --- | --- | --- |
|  |  |  |  |  |  |  |  |  |  |  |  |  | **1950-2000** | **2020** | **2050** | **2080** |  |  |
| N1 | 1870 | Mar Menor | Possible nest | 37.771 | -0.786 | - | 1870 | - | - | - | - | Good | 35,9 | (+)10 | (+)5 | (+)5 | - | Carreras et al. 2018 |
| N2 | 1990 | Ebro delta | Dead embryo | 40.655 | 0.789 | - | 09/1990 | - | - | - | - | Moderate | 21,7 | (+)10 | (+)10 | (+)10 | - | Carreras et al. 2018 |
| N3 | 2001 | Vera | Full nest | 37.222 | -1.802 | - | 27/07/2001 | 97 | 43,3 | 58 | 65_22_72_10 | Good | 35,9 | (+)10 | (+)10 | (+)5 | CC-A3.1 | Carreras et al. 2018 |
| N13 | 2006 | Puzol | Full nest | 39.607 | -0.265 | - | 11/08/2006 | >78 | 36,8 | >50 | - | Moderate | 21,7 | (+)10 | (+)10 | (+)10 | CC-A2.1 | Carreras et al. 2018 |
| N14 | 2006 | Premià de Mar | Full nest | 41.488 | 2.357 | - | 27/10/2006 | 82 | 68,3 | - | - | Marginal | 14,5 | (+)10 | (+)10 | (+)10 | CC-A1.1 | Carreras et al. 2018 |
| N21 | 2011 | Malgrat de Mar | Full nest | 41.645 | 2.752 | - | 01/10/2011 | 134 | 70,8 | - | - | Marginal | 14,5 | (+)10 | (+)10 | (+)10 | CC-A2.1 | Carreras et al. 2018 |
| N27 | 2014 | Alicante | Full nest | 38.376 | -0.409 | 30/06/2014 | 28/08/2014 | 131 | 79 | 59 | 57_12_52_3 | Moderate | 21,7 | (+)10 | (+)10 | (+)10 | CC-A20.1 | Carreras et al. 2018 |
| N28 | 2014 | Tarragona | Full nest | 41.119 | 1.276 | 31/08/2014 | *-* | 89 | 0 | - | - | Marginal | 14,5 | (+)10 | (+)10 | (+)10 | CC-A3.1 | Carreras et al. 2018 |
| N29 | 2014 | Tarragona | Full nest | 41.127 | 1.296 | - | 30/10/2014 | 58 | - | - | - | Marginal | 14,5 | (+)10 | (+)10 | (+)10 | CC-A2.1 | Carreras et al. 2018 |
| N32 | 2015 | Torrevieja | Full nest | 38.015 | -0.653 | 31/07/2015 | 09/10/2015 | 85 | 32,5 | 53,5 | 89_87_99_99 | Moderate | 21,7 | (+)10 | (+)10 | (+)10 | CC-A2.1 | Carreras et al. 2018 |
| N33 | 2015 | Pulpí | Full nest | 37.345 | -1.684 | 17/07/2015 | 09/09/2015 | 81 | 52,9 | 69 | 5_0_0_0 | Good | 35,9 | (+)10 | (+)10 | (+)10 | CC-A2.1 | Carreras et al. 2018 |
| SP01 | 2016 | Les Palmeres (Sueca) | Full nest | 39.256 | -0.262 | 03/07/2016 | 05/09/2016 | 88 | 54 | 63 | 26_0_4_0 | Excellent | 76.9 | (+)5 | (-)5 | (-)10 | CC-A2.-1 | Present study |
| SP02 | 2017 | Migjorn (Peñiscola) | Hatchlings on beach | 40.358 | 0.403 | - | 11/10/2017 | - | - | - | - | Moderate | 21,7 | (+)10 | (+)10 | (+)10 | CC-A1.-1 | Present study |
| SP03 | 2018 | Sant Simó (Mataró) | Full nest | 41.541 | 2.459 | 15/06/2018 | 08/08/2018 | 173 | 56,1 | 54 | 87_85_98_99 | Marginal | 14,5 | (+)10 | (+)10 | (+)10 | - | Present study |
| SP04 | 2018 | La Descàrrega (Premià de Mar) | Full nest | 41.486 | 2.349 | 01/08/2018 | 28/09/2018 | 62 | 93,1 | 58 | 65_22_72_10 | Marginal | 14,5 | (+)10 | (+)10 | (+)10 | CC-A3.1 | Present study |
| SP05 | 2018 | Vilafortuny (Cambrils) | Full nest | 41.069 | 1.099 | - | 16/09/2018 | 112 | 80,4 | - | - | Marginal | 14,5 | (+)10 | (+)10 | (+)10 | CC-A2.-1 | Present study |
| SP06 | 2018 | Ardiaca  (Cambrils) | Hatchlings on beach | 41.054 | 1.028 | - | 24/09/2018 | - | - | - | - | Marginal | 14,5 | (+)10 | (+)10 | (+)10 | - | Present study |
| SP07 | 2019 | Del Serradal (Castellón de la Plana) | Full nest | 40.005 | 0.032 | 13/07/2019 | 14/09/2019 | 112 | 65 | 63 | 26_0_4_0 | Moderate | 21,7 | (+)10 | (+)10 | (+)10 | CC-A31.-1 | Present study |
| SP08 | 2019 | D’en Bossa  (Sant Jordi de ses Salines) | Full nest | 38.884 | 1.406 | 25/07/2019 | 10/09/2019 | 58 | 65,5 | 46 | 100_100_100_100 | Good | 21,7 | (+)10 | (+)10 | (+)10 | CC-A2.-1 | Present study |
| SP09 | 2019 | Calblanque (Cartagena) | Full nest | 37.598 | -0.750 | 28/07/2019 | 18/09/2019 | 69 | 30,4 | 52 | 93_96_100_100 | Excellent | 35,9 | (+)10 | 0 | (-)10 | CC-A2.-1 | Present study |
| SP10 | 2019 | D’es Cavallet  (Sant Francesc de s’Estany) | Full nest | 38.841 | 1.403 | 29/07/2019 | - | 102 | 0 | - | - | Marginal | 21,7 | (+)10 | (+)10 | (+)10 | - | Present study |
| SP11 | 2019 | Castelldefels (Castelldefels) | Hatchlings on beach | 41.264 | 1.957 | - | 06/10/2019 | - | - | - | - | Marginal | 14,5 | (+)10 | (+)10 | (+)10 | CC-A31.-1 | Present study |

Supplementary table 1. Sporadic nests of loggerhead turtle in the Spanish coast from 1870 to 2019 and data collected. *Eg:* number of eggs ; *Hs*: hatchling success (%); *Id*: Incubation duration (days); *Of:* percentage of female offspring using *Id* and calculated from different models^1–4^; Habitat: predicted habitat suitability for loggerhead turtles as estimated from MaxEnt models for the Mediterranean region^5^; Predicted Hs: Predicted hatchling success at different years as predicted in published models^6^, the baseline value corresponds to the period between 1950 and 2000 and the rest of values indicate the expected percentage of increase or decrease respect the baseline values.

| **Attempt code** | **Location** | **Latitude [°N]** | **Longitude [°E]** | **Year** | **Attempt type** |
| --- | --- | --- | --- | --- | --- |
| A1 | Delta del Ebro | - | - | 1972-73 | Track |
| ES_C01 | Girona | 41.673 | 2.793 | 2014 | Female |
| ES_C02 | Barcelona | 41.613 | 2.653 | 2014 | Female |
| ES_C03 | Almeria | 36.939 | -1.934 | 2014 | Female |
| ES_C04 | Ibiza | 38.098 | 1.5540 | 2015 | Female |
| ES_C05 | Ibiza | 38.983 | 1.5347 | 2015 | Female |
| ES_C06 | Ibiza | 38.983 | 1.534 | 2015 | Female |
| ES_C07 | Murcia | 37.672 | -0.728 | 2015 | Female |
| ES_C08 | Murcia | 37.4018 | -1.591 | 2015 | Female |
| ES_C09 | Murcia | 37.599 | -0.7502 | 2016 | Track |
| A2 | Castelldefels | 41.281 | 2.088 | 2016 | Female |
| ES_C10 | Almeria | 37.247 | -1.768 | 2016 | Female |
| ES_C11 | Alicante | 38.355 | -0.431 | 2016 | Female |
| ES_C12 | Ibiza | 38.901 | 1.421 | 2017 | Female |
| ES_C13 | Barcelona | 41.585 | 2.580 | 2017 | Female |
| ES_C14 | Alicante | 38.054 | -0.651 | 2017 | Track |
| ES_C15 | Murcia | 37.700 | -0.739 | 2017 | Female |
| ES_C16 | Murcia | 37.599 | -0.750 | 2017 | Female |
| ES_C17 | Murcia | 37.599 | -0.7502 | 2017 | Track |
| ES_C18 | Amposta | 40.654 | 0.801 | 2017 | Track |
| ES_C19 | Murcia | 37.598 | -0.751 | 2017 | Track |
| ES_C20 | Murcia | 37.680 | -0.732 | 2017 | Female |
| ES_C21 | Valencia | 39.513 | -0.319 | 2018 | Female |
| ES_C22 | Castellón | 40.042 | 0.060 | 2018 | Female |
| ES_C23 | Altea | 38.630 | -0.006 | 2018 | Female |
| ES_C24 | Orihuela | 37.917 | -0.719 | 2018 | Female |
| ES_C25 | Alicante | 37.871 | -0.754 | 2018 | Female |
| ES_C26 | Barcelona | 41.284 | 2.0960 | 2018 | Female |
| ES_C27 | Murcia | 37.597 | -0.762 | 2019 | Track |
| ES_C28 | Tarragona | 40.68 | 0.852 | 2019 | Track |
| ES_C29 | Murcia | 37.718 | -0.7407 | 2019 | Track |
| ES_C30 | Murcia | 37.718 | -0.7407 | 2019 | Track |

Supplementary Table S2. List of sporadic loggerhead turtle nesting attempts on the Mediterranean Spanish coast from 1972 to 2019. Female indicates that an adult female was found on the beach but no related nest was found. Track indicates that turtle tracks were found on the beach but without evidences of a nest. Data obtained from Hochscheid et al. (2022) ^7^, except A1^8^ and A2 (present study).

| **Nest Code** | **Beach (Locality)** | **Individual Code** | **Ho** | **Raw reads** | **Filtered reads** | **Mapped reads [%]** | **Number of loci** |
| --- | --- | --- | --- | --- | --- | --- | --- |
| SP01 | Les Palmeres (Sueca) | SP01_1 | 0.252 | 5,381,655 | 4,609,524 | 95.4 | 2290 |
|  |  | SP01_2 | 0.260 | 3,268,581 | 2,811,413 | 95.5 | 2277 |
| SP02 | Migjorn (Peñiscola) | SP02_1 | 0.257 | 6,380,033 | 5,465,033 | 95.5 | 2291 |
|  |  | SP02_2 | 0.240 | 5,927,855 | 5,169,399 | 95.0 | 2290 |
| SP04 | La Descàrrega (Premià de Mar) | SP04_1 | 0.252 | 6,386,869 | 4,126,609 | 95.5 | 2273 |
|  |  | SP04_2 | 0.250 | 7,474,811 | 6,109,951 | 95.3 | 2291 |
|  |  | SP04_3 | 0.267 | 9,888,986 | 8,091,694 | 95.6 | 2290 |
|  |  | SP04_4 | 0.251 | 7,380,964 | 5,910,627 | 94.4 | 2290 |
|  |  | SP04_5 | 0.257 | 8,538,495 | 6,761,385 | 95.5 | 2291 |
|  |  | SP04_6 | 0.255 | 8,279,236 | 7,052,296 | 95.7 | 2291 |
|  |  | SP04_7 | 0.261 | 8,698,409 | 7,428,122 | 95.7 | 2291 |
|  |  | SP04_8 | 0.263 | 4,320,601 | 3,885,076 | 95.7 | 2281 |
| SP05 | Vilafortuny (Cambrils) | SP05_1 | 0.281 | 3,823,190 | 3,319,194 | 91.3 | 2289 |
|  |  | SP05_2 | 0.324 | 8,279,366 | 6,880,997 | 74.4 | 2291 |
|  |  | SP05_3 | 0.259 | 6,473,971 | 5,672,384 | 95.7 | 2290 |
|  |  | SP05_4 | 0.251 | 5,258,447 | 3,512,435 | 57.9 | 2221 |
| SP07 | Del Serradal (Castellón de la Plana) | SP07_1 | 0.251 | 4,070,232 | 2,698,674 | 95.9 | 2288 |
|  |  | SP07_2 | 0.269 | 4,784,831 | 3,797,112 | 95.7 | 2291 |
|  |  | SP07_3 | 0.264 | 3,865,348 | 3,017,269 | 95.5 | 2290 |
|  |  | SP07_4 | 0.280 | 6,083,117 | 5,090,768 | 94.3 | 2202 |
|  |  | SP07_5 | 0.259 | 4,316,300 | 3,473,349 | 95.9 | 2288 |
|  |  | SP07_6 | 0.249 | 4,654,665 | 3,103,020 | 95.7 | 2290 |
|  |  | SP07_7 | 0.263 | 5,030,768 | 4,097,368 | 94.2 | 2006 |
| SP08 | D’en Bossa  (Sant Jordi de ses Salines) | SP08_1 | 0.265 | 4,660,832 | 3,439,416 | 95.6 | 2291 |
|  |  | SP08_2 | 0.252 | 5,754,139 | 4,527,912 | 95.6 | 2291 |
|  |  | SP08_3 | 0.269 | 4,914,376 | 4,092,821 | 93.9 | 2079 |
|  |  | SP08_4 | 0.264 | 4,511,422 | 3,782,799 | 93.8 | 2006 |
|  |  | SP08_5 | 0.265 | 4,244,291 | 3,288,598 | 95.4 | 2289 |
|  |  | SP08_6 | 0.299 | 6,187,107 | 5,254,976 | 93.8 | 2200 |
|  |  | SP08_7 | 0.277 | 4,768,676 | 4,047,806 | 93.8 | 2030 |
| SP09 | Calblanque (Cartagena) | SP09_1 | 0.255 | 5,549,998 | 4,524,128 | 95.9 | 2291 |
|  |  | SP09_2 | 0.251 | 5,175,547 | 3,967,852 | 95.7 | 2291 |
|  |  | SP09_3 | 0.263 | 5,065,870 | 4,032,402 | 93.0 | 2042 |
|  |  | SP09_4 | 0.279 | 5,231,907 | 3,950,012 | 94.0 | 2002 |
|  |  | SP09_5 | 0.258 | 4,288,713 | 3,373,797 | 95.5 | 2287 |
|  |  | SP09_6 | 0.256 | 3,950,197 | 3,693,181 | 95.6 | 2289 |
|  |  | SP09_7 | 0.259 | 4,491,761 | 3,579,951 | 95.7 | 2291 |
| SP11 | Castelldefels (Castelldefels) | SP11_1 | 0.246 | 3,039,933 | 2,615,349 | 95.0 | 2282 |
|  |  | SP11_2 | 0.244 | 6,505,721 | 5,299,574 | 95.0 | 2291 |
|  |  | SP11_3 | 0.248 | 5,381,471 | 4,306,982 | 95.9 | 2291 |
|  |  | SP11_4 | 0.245 | 4,270,152 | 3,544,631 | 95 | 2290 |
|  |  | SP11_5 | 0.267 | 2,426,253 | 1,297,965 | 95.8 | 2073 |
|  |  | SP11_6 | 0.248 | 5,468,171 | 3,958,123 | 95.9 | 2291 |
|  |  | SP11_7 | 0.247 | 5,549,441 | 4,455,992 | 95 | 2291 |
|  |  | SP11_8 | 0.267 | 6,478,317 | 5,353,343 | 95.9 | 2291 |

Supplementary table 3. Individuals genotyped and associated genomic data of the 45 individuals. *Ho*: individual observed heterozygosity; Filtered reads: number of trimmed sequences; Mapped reads: percentage of reads mapped against the reference genome; Number of loci: number of polymorphic loci per individual.

**REFERENCES OF THE SUPLEMENTARY TABLES**

1. Mrosovsky, N., Kamel, S., Rees, A. F. & Margaritoulis, D. Pivotal temperature for loggerhead turtles (*Caretta caretta*) from Kyparissia Bay, Greece. *Can. J. Zool.* **80,** 2118–2124 (2002).

2. Fuller, W. J. *et al.* Importance of spatio-temporal data for predicting the effects of climate change on marine turtle sex ratios. *Mar. Ecol. Prog. Ser.* **488,** 267–274 (2013).

3. Mrosovsky, N., Baptistotte, C. & Godfrey, M. H. Validation of incubation duration as an index of the sex ratio of hatchling sea turtles. *Can. J. Zoo.l* **77,** (1999).

4. Marcovaldi, M. A., Godfrey, M. H. & Mrosovsky, N. Estimating sex ratios of loggerhead turtles in Brazil from pivotal incubation durations. *Can. J. Zool.* **75,** 755–770 (1997).

5. Pike, D. A. Climate influences the global distribution of sea turtle nesting. *Glob. Ecol. Biogeogr.* **22,** 555–566 (2013).

6. Pike, D. A. Forecasting the viability of sea turtle eggs in a warming world. *Glob. Chang. Biol.* **20,** 7–15 (2014).

7. Hochscheid, S. *et al.* Nesting range expansion of loggerhead turtles in the Mediterranean: Phenology, spatial distribution, and conservation implications. *Glob. Ecol. Conserv.* **38,** e02194 (2022).

8. Salvador, F. Naturalista in situ, recogido en el II Congreso Luso-Español de Herpetología. (1987).
